# Musculotendinous Stiffness of Triceps Surae, Maximal Rate of Force Development, and Vertical Jump Performance

**DOI:** 10.1155/2015/797256

**Published:** 2015-01-29

**Authors:** Tarak Driss, Daniel Lambertz, Majdi Rouis, Hamdi Jaafar, Henry Vandewalle

**Affiliations:** ^1^CeRSM (EA 2931), Equipe de Physiologie, Biomécanique et Imagerie du Mouvement, UFR STAPS, Université Paris Ouest Nanterre La Défense, 200 avenue de la République, 92000 Nanterre, France; ^2^CNRS UMR 7338, Biomécanique et Bioingénierie, Université de Technologie de Compiègne, 60205 Compiègne, France; ^3^Laboratoire de Physiologie, UFR de Santé, Médecine et Biologie Humaine, Université Paris XIII, rue Marcel Cachin, 93017 Bobigny, France

## Abstract

The relationships between ankle plantar flexor musculotendinous stiffness (MTS) and performance in a countermovement vertical jump (CMJ) and maximal rate of torque development (MRTD) were studied in 27 active men. MTS was studied by means of quick releases at 20 (*S*_0.2_), 40 (*S*_0.4_), 60 (*S*_0.6_), and 80% (*S*_0.8_) of maximal voluntary torque (*T*_MVC_). CMJ was not correlated with strength indices but was positively correlated with MRTD/BM, *S*_0.4_/BM. The slope *α*_2_ and intercept *β*_2_ of the torque-stiffness relationships from 40 to 80% *T*_MVC_ were correlated negatively (*α*_2_) and positively (*β*_2_) with CMJ. The different stiffness indices were not correlated with MRTD. The prediction of CMJ was improved by the introduction of MRTD in multiple regressions between CMJ and stiffness. CMJ was also negatively correlated with indices of curvature of the torque-stiffness relationship. The subjects were subdivided in 3 groups in function of CMJ (groups H, M, and L for high, medium, and low performers, resp.). There was a downward curvature of the torque-stiffness relationship at high torques in group H or M and the torque-stiffness regression was linear in group L only. These results suggested that torque-stiffness relationships with a plateau at high torques are more frequent in the best jumpers.

## 1. Introduction

Since its presentation in 1921 by Sargent as “the physical test of a man” [1], vertical jump is often used as field or laboratory test. The interest of vertical jump test as power tests was confirmed by the significant correlations between maximal power measured on a cycle ergometer (*P*_max⁡_) and vertical jump [2–5]. However, in these studies, the prediction of vertical jump height with countermovement (CMJ) or squat jump (SJ) from *P*_max⁡_ is not accurate which suggested that the performances in vertical jump tests also depend on other factors than the maximal power of the lower limbs.

The rise in the force exerted on the ground depends on the rate of force development by the lower limb muscles. Therefore, CMJ should be positively correlated with maximal rate of torque development (MRTD) by these muscles. As the rate of force development depends on the series elastic component [6], CMJ should also be positively correlated with musculotendinous stiffness (MTS) of the lower limb muscles. The performances in vertical jump tests have been correlated with MTS of the quadriceps muscle in ultrasonography studies [7–10]. Kubo et al. [10] reported a negative correlation between the elastic properties of tendon structures of the vastus lateralis and the difference between CMJ and SJ. On the other hand, Bojsen-Møller et al. [7] reported a positive correlation between tendon stiffness of the vastus lateralis and the maximal height of a countermovement jump. Most of the power produced by the hip and knee extensors is transmitted to the foot at the articular surface of the talus. The sum of the moments of the forces exerted by the different plantar flexor muscles is equal to the moment of ground reaction force around the ankle rotation centre (the inertia and angular acceleration of the foot are considered as negligible). The triceps surae is the main plantar flexor. Therefore, the rise in the force exerted on the ground depends on the rise in the force exerted by the calcaneal tendon on the foot although the triceps surae is not the major source of power during vertical jump [11–13]. Some studies in the literature suggested that the performances in speed and power exercises depend on MTS of the triceps surae [14]. Moreover, an increase in tendon compliance might improve elastic energy storage and utilization [15]. This storage and this utilization of elastic potential energy should be more important in the calcaneal tendon, which is several times longer than the quadriceps tendon. Whether the correlation between CMJ and MTS is positive or negative is still under debate, and the discrepancies between these studies probably depend on the protocols. Moreover, other studies indicated that the influence of MTS on jumping performance was negligible [15, 16].

In the present investigation, it was hypothesized that ankle plantar flexor MTS and maximal rate of torque development (MRTD) were factors influencing performance in CMJ. The relationship between countermovement vertical jump performance and ankle plantar flexor MTS was estimated from the results of quick releases during plantar flexion and CMJ.

## 2. Materials and Methods

### 2.1. Subjects

Twenty-seven male subjects participated in this study (22.41 ± 1.98 years). They were all healthy active male physical education students. In order to study the relation between MRTD, MTS, and CMJ, they were subdivided in 3 groups (3 × 9) in function of their results in the vertical jump test: high (group H), medium (group M), and low performers (group L). Body mass (BM) and body height (BH) are presented in Table 1. All procedures were explained to the participants, a written consent form was completed before the study procedures were started, and the study was carried out according to the guidelines of the Declaration of Helsinki. The experimental protocol was approved by the Committee of Hygiene, Safety and Ethics of the University of Compiègne.

### 2.2. Testing Device

#### 2.2.1. Ankle Ergometer

**Figure 1:**
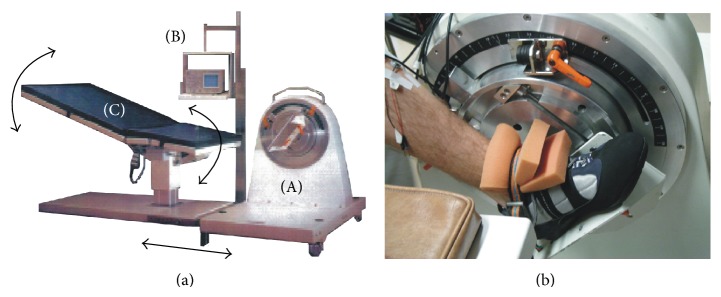
(a) The ankle ergometer system. (A) Actuator, with its power supply unit, angular displacement, angular velocity, and torque transducers, and its associated electronics; (B) driving unit controlled by a personal computer; (C) adjustable table. (b) Foot strapping on the actuator.

Ankle plantar flexor MTS was studied by means of the ankle ergometer designed by the University of Technology of Compiègne (France) for the measurement of ankle plantar flexor MTS before and after long-term spaceflights [17]. This ankle ergometer (Figure 1) consists of two main units: (1) a power unit that contained the actuator, its power supply unit, angular displacement, angular velocity and torque transducers, and its associated electronics; (2) a driving unit controlled by a personal computer equipped with a 12-bit analog-to-digital board; (3) adjustable table. Angular displacement was measured with an optical digital sensor, and angular velocity was captured from a resolver bound to the rotor, except for velocities >15.7 rad·s^−1^ that required a tachometer. Torque was obtained by means of a strain gauge torque transducer. All mechanical data were sampled at 1 kHz. A dual beam oscilloscope gave the subjects visual feedback about the procedure in progress.

#### 2.2.2. Vertical Jump Device

Maximal countermovement vertical jump (CMJ) was performed with the device described by Vandewalle et al. [3], Driss et al. [4], and Rouis et al. [5]. CMJ corresponded to the distance between the body height of the subjects and the level reached by the head at the peak of the jump.

### 2.3. Experimental Protocol

#### 2.3.1. Measurement of Torque and Stiffness

The subjects were comfortably lying on an adjustable table with the left foot attached rigidly to the actuator of the ankle ergometer by a cyclist shoe with a rigid sole (Figure 1(b)). Different sizes were available to adjust the shoe to the foot. The lateral malleolus coincided with the axis of rotation of the footplate. The knee was extended to 120° (full extension = 180°) and the ankle was placed at 90° (i.e., the neutral position). The shoulders were maintained by special shoulder holders, so that the subjects could not move. The left thigh was fastened to the table by a large strap above the patella.

In first test, the maximal torque under isometric conditions was determined during a maximal voluntary contraction of the plantar flexors. The subjects were instructed to develop a maximal contraction as fast as possible against the actuator and to maintain this contraction during 2 seconds. Three contractions with 90-second recovery intervals were carried out, and the best performance was considered as the torque at maximal voluntary contraction (*T*_MVC_).

Secondly, quick release tests were performed. As in isolated muscle, the aim of this test is to determine the characteristics of the so-called series elastic component (SEC) (i.e., MTS). Quick release movements from the neutral position were achieved by sudden releasing of the moving parts of the device while the subjects were instructed to maintain submaximal plantar flexion torques of around 20% 40%, 60%, or 80% of *T*_MVC_. The required torques and the actual torques were displayed on an oscilloscope during these instructions. Three successive contractions were performed at 20, 40, 60, and 80% *T*_MVC_ in this order for all the subjects. To prevent muscle fatigue, one-minute resting periods between the different trials were observed.

Finally, the maximal rate of torque development (MRTD) was measured according to Sahaly et al. [18]. The subjects were instructed to produce the most rapid force production (i.e., to concentrate on the fastest contraction without concern for achieving maximal voluntary force). The subjects were not given any feedback on their performances but were encouraged to produce their maximal effort. Four contractions with 90-second recovery intervals were performed and MRTD was considered as the greatest value achieved in the four contractions. All the torque measurements were sampled at 1 kHz.

#### 2.3.2. Vertical Jump

The vertical jump test was a countermovement jump which is probably a much more natural jumping movement than a squat jump and one advantage of a countermovement is that the leg muscles attain a higher level of activation and force before they start to shorten [19]. As in the protocol used in the study by Vandewalle et al. [3], the countermovement was associated with an arm swing. The subjects performed 2 or 3 jumps with 10 to 15 s recovery between these trials. Thereafter, the subjects had 2-minute recovery before jumping again. The height of the stick was increased after each trial providing they were able to hit it with their vertex. When the subjects were only able to brush the stick, the height was increased by 0.5 cm only. The maximal height corresponded to the highest height before three consecutive unsuccessful trials corresponding to a 0.5 cm increase. Approximately 10 jumps were performed and the highest result was noted.

The reliability of CMJ performance is high when measured with this device and this protocol. In a study on 31 subjects (unpublished personal data), the intraclass coefficient was 0.98, the test-retest coefficient of correlation was 0.978, and the differences between the first and second sessions ranged only from −1 to 1 cm in 21 from the 31 subjects.

### 2.4. Data Processing

#### 2.4.1. Quick Release Tests

In quick and control release methods, MTS is estimated by recording the decline in moment as a function of joint rotation and is expressed in newton-meters per radian. These methods do not require knowledge about the lengths of the tendon moment arms of the individual muscles. However, the translation of the rotational measure of MTS into linear measures of MTS of the individual muscles (the different ankle plantar flexors) needs information about tendon dimensions, tendons moment arms, and force sharing. In quick release methods, the decline in moment is measured from the decline in acceleration. MTS is calculated as the ratio *S* between variations in angular acceleration (Θ′′) and angular displacement (Θ) within a time lapse of 20 ms (i.e., when elastic elements are supposed to recoil), as expressed by

(1) $$\begin{array}{c} S=\frac{{\Delta I\cdot \Theta }^{\prime \prime }}{\Delta \Theta }=\frac{I\cdot \Delta \Theta^{\prime \prime }}{\Delta \Theta }. \end{array}$$

In this equation, inertia is assumed to be constant. This can be verified easily by considering the transition between the static phase and the dynamic phase. At this moment static torque (*T*) equals dynamic torque and acceleration is maximal:

(2) $$\begin{array}{c} I\cdot \Theta_{\max}^{\prime \prime }=T. \end{array}$$

MTS characteristics were measured at the very beginning of the quick release movement (i.e., before any reflex activation; e.g., unloading reflex [20]). Then, the value of *S* was related to the corresponding isometric torque initially exerted by the subject. The *T*-*S* relationship is often approximated by a linear relationship [21, 22]:

(3a) $$\begin{array}{c} S=\frac{\Delta T}{\Delta \Theta }=\alpha T+\beta . \end{array}$$

**Figure 2:**
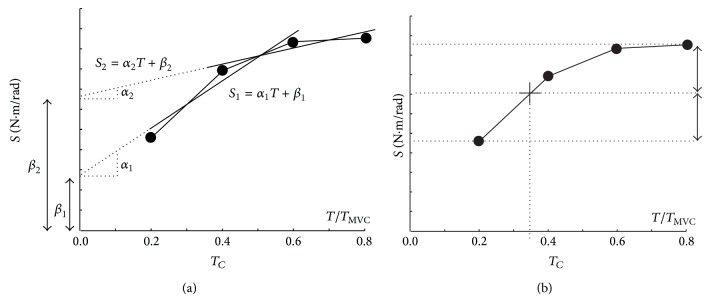
(a) Determination of slopes *α*_1_, *α*_2_ and intercepts *β*_1_, *β*_2_ of the torque-stiffness relationship. Torque (*T*) is expressed as a fraction of the torque produced during a maximal voluntary contraction in isometric mode (*T*_MVC_). (b) Determination of the curvature index *T*_*C*_ equal to the torque corresponding to half of the difference in stiffness at 0.4 and 0.8 *T*_MVC_.

This slope is an index of stiffness that has the advantage to be independent of the required torque level and to avoid the use of MVC or cross-sectional measurements for normalizing MTS [21, 22]. In contrast to these previous studies mainly performed on nonathletic subjects, a downward inflection of the *T*-*S* relationship at high torque has been observed in several subjects in the present study. Different models of curvilinear relationship between *T* and *S* (power, logarithmic, exponential…) have been tested but none of them could fit the experimental data in all the subjects. Indeed, the value of *S* extrapolated for zero torque must be positive but *S* at zero torque was negative in some subjects. Moreover, a curvilinear model could not accurately fit the experimental data in the subjects whose *T*-*S* relationships were linear and the difference between observed and predicted data was high in some subjects. Therefore, the *T*-*S* curve was approximated by two linear segments (Figure 2):

(3b) $$\begin{array}{c} S_{1}=\alpha_{1}T+\beta_{1}\;\text{for}\;T\;\text{ranging}\;\text{from}\;20\;\text{to}\;60\%\;\text{MVC}, \end{array}$$

(3c) $$\begin{array}{c} S_{2}=\alpha_{2}T+\beta_{2}\;\text{for}\;T\;\text{ranging}\;\text{from}\;40\;\text{to}\;80\%\;\text{MVC}. \end{array}$$

Consider *α*_1_ = *α*_2_ and *β*_1_ = *β*_2_ and ratio *α*_2_/*α*_1_ = 1 if the *T*-*S* relationship is linear. The *α*_2_/*α*_1_ ratio was used as an index of curvature of the *T*-*S* relationship. Another empirical curvature index (*T*_*C*_) was computed as equal to the torque corresponding to the half of the difference in stiffness at 0.4 and 0.8 *T*_MVC_. Perfectly linear relationships correspond to values of *T*_*C*_ equal to 0.5 and curvilinear relationship corresponds to values lower than 0.5.

In spite of the display of torque output on an oscilloscope, the actual torques corresponding to 20, 40, 60, and 80% *T*_MVC_ instructions were not exactly equal to the required torques. Therefore, the values of *S* which corresponded exactly to 20, 40, 60, and 80% *T*_MVC_ (*S*_0.2_, *S*_0.4_, *S*_0.6_, and *S*_0.8_) for each subject were computed by linear interpolation (or extrapolation) of the experimental individual data. The values of *T* and *S* used in these interpolations were the averages of the three contractions corresponding to a given instruction (20, 40, 60, or 80% *T*_MVC_).

The value of *S* corresponding to a torque equal to 100 N·m (*S*100) was computed by linear interpolation of the experimental torque-stiffness data to compare MTS in the present study with the data in previous studies that used the quick release methods for the assessment of triceps surae stiffness.

#### 2.4.2. Computation of MRTD

The rate of torque development (RTD in N m s^−1^) was computed as the difference in torque between times *t* (*T*_*t*_) and *t* + 20 ms (*T*_*t*+20_) [18]:

(4) $$\begin{array}{c} \text{RTD}=\frac{\left(T_{t+20}-T_{t}\right)}{0.02}. \end{array}$$

The RTD was calculated from *t* = 0 to the end of the torque measurement for each value of *T*_*t*_. MRTD corresponded to the highest value of RTD [18].

#### 2.4.3. Expression of the Data

The value of CMJ is probably independent of body mass (BM^0^) as suggested by the results of an allometric study [23]. Torque has the dimension of the product of a force and a distance (N·m). Consequently, the torque exerted by a muscle group should be proportional to body mass (BM^1^) as force and arm lever are proportional to BM^2/3^ and BM^1/3^, respectively. The dimensions of *S*_0.2_, *S*_0.4_, *S*_0.6_, and *S*_0.8_ are equal to torque·rad^−1^, that is, BM^1^·rad^−1^. With other things being equal, the values of *S*_0.2_, *S*_0.4_, *S*_0.6_, and *S*_0.8_ should depend on BM^1^. Therefore, the values of* S*_0.2_/BM,* S*_0.4_/BM,* S*_0.6_/BM, and* S*_0.8_/BM should be independent of body mass (BM^1^ rad^−1^·BM^−1^ = BM^0^·rad^−1^ = rad^−1^). Consequently, the value of *S* corresponding to the different torques (20, 40, 60, and 80% *T*_MVC_) was normalized to body mass (*S*_0.2_/BM,* S*_0.4_/BM,* S*_0.6_/BM, and* S*_0.8_/BM) when CMJ was correlated with stiffness.

When normalized to *T*_MVC_, the value of *S* corresponding to the different torques (20, 40, 60, and 80% *T*_MVC_) is a dimensionless variable (N·m·rad^−1^·N^−1^·m^−1^ = N^0^·m^0^·rad^−1^ = rad^−1^). Therefore, *S* was expressed as* S*_0.2_/*T*_MVC_,* S*_0.4_/*T*_MVC_,* S*_0.6_/*T*_MVC_, and* S*_0.8_/*T*_MVC_ for the comparisons of the torque-stiffness relationships in the different groups. As stiffness indices and for the same reasons, MRTD was related to body mass (N·m/s·BM) or *T*_MVC_ (s^−1^) to study the relationship between CMJ and the rate of torque development.

### 2.5. Statistics

The effect of MTS or MRTD upon CMJ was studied by means of the correlations between CMJ and the different indices of stiffness and rate of torque development in the whole group (*n* = 27). The comparisons of the results in groups H, M, and L for the anthropometric data, strength indices (*T*_MVC_, *T*_MVC_/BM, MRTD/BM, MRTD/*T*_MVC_), and stiffness indices (*S*_0.4_/BM and* S*_0.4_/*T*_MVC_,* S*100, *α*_1_, *α*_2_, *β*_1_, *β*_2_, and ratio *α*_2_/*α*_1_) were tested with a one-way ANOVA and a post hoc Bonferroni *t*-test. The differences between *α*_1_ and *α*_2_ or between *β*_1_ and *β*_2_ were tested with a two-way ANOVA with repeated measures and a post hoc Bonferroni *t*-test.

The linearity of the stiffness-torque relationship was verified with a lack-of-fit test based on variance analysis:

(5) $$\begin{array}{c} F=\frac{\left(A/{\text{dof}}_{A}\right)}{\left(B/{\text{dof}}_{B}\right)}, \end{array}$$

where *A* is sum of squares due to lack-of-fit of the model; *B* is sum of squares due to pure error; dof_*A*_ is degree of freedom of *A* = *n* − *p*; dof_*B*_ is degree of freedom of *B* = *N* − *n*; *N* is total number of stiffness values; *n* is number of torque values = 4; *p* is number of parameters in the model = 2; dof_*A*_ = 2; dof_*B*_ = 104 for the torque-stiffness relationship corresponding to all the participants and dof_*B*_ = 32 for the torque-stiffness relationships corresponding to groups H, M, and L.

All statistical analyses were conducted at *P* < 0.05. Values were presented as mean ± SD in tables, and as mean ± standard error in figures. Statistical analyses were carried out using Sigma-Stat and Sigma-Plot Software (Jandel Scientific, Germany).

## 3. Results

There was no significant difference in BM and BH between the different groups (Table 1). In addition, CMJ was independent of BM and BH (0.031 < *r* < 0.133; *P* > 0.05).

### 3.1. Ankle* T-S* Relationship

**Figure 3:**
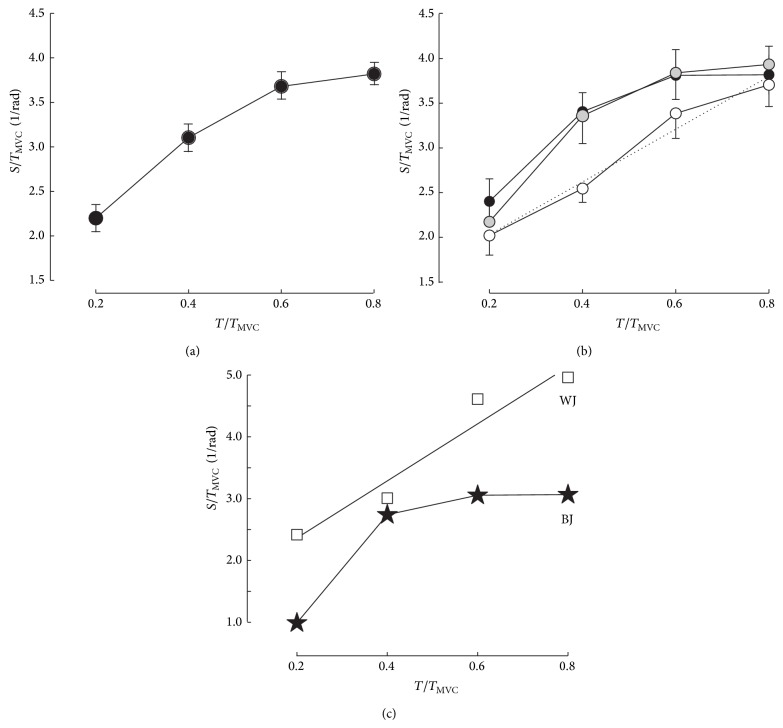
Relationships between torque (abscissa) in fraction of the torque produced during a maximal voluntary contraction (*T*_MVC_) and stiffness normalized to *T*_MVC_. In (a), all the subjects (*n* = 27). In (b), comparison of groups H (black dots), M (grey dots), and L (empty circles). In (c), the results of the best performer in countermovement jump (BJ) are compared with those of the worst performer (WJ).

The *T*-*S* relationships are presented in Figure 3(a) for the whole group (*n* = 27) and in Figure 3(b) for groups H, M, and L. The different indices of stiffness are given in Table 2. The differences between group M and the other groups were significant for* S*_0.4_/*T*_MVC_ (Table 2 and Figure 3(b)). For* S*_0.4_/BM, it was the differences between group H and the other groups that were significant (Table 2).

There was no significant difference between groups H, M, and L for slope *α*_1_. In contrast, *α*_2_ was significantly higher in group L when compared to group H or M (*P* ≤ 0.041; Table 2). The differences between slopes *α*_2_ and *α*_1_ (Table 2) were significant in the whole group (*P* < 0.001), groups H (*P* < 0.001) and M (*P* < 0.001) but not in group L (*P* = 0.216). Slope *β*_2_ was significantly lower in group L when compared to group H or M (*P* ≤ 0.016; Table 2).

The *T*-*S* relationship of the whole group was significantly different (*F*_2,104_ = 7.177; *P* < 0.001) from a linear relationship. The lack-of-fit tests indicated that the torque-stiffness relationships were significantly different from a linear relationship for groups H (*F*_2,32_ = 13.7; *P* < 0.01) and M (*F*_2,32_ = 5.40; *P* = 0.05) but not for group L (*F*_2,32_ = 1.02; *P* > 0.05).

### 3.2. CMJ and Isometric Strength Indices

The indices of torque and rate of torque development in absolute values (*T*_MVC_, MRTD) and in values related to body mass (*T*_MVC_/BM, MRTD/BM) or to *T*_MVC_ (MRTD/*T*_MVC_) are given in Table 1. There was no significant difference in *T*_MVC_ or *T*_MVC_/BM between groups H, M, and L. The value of CMJ was not significantly correlated (*r* < 0.227, *P* > 0.255) with strength indices (*T*_MVC_ or *T*_MVC_/BM).

The differences in MRTD or MRTD/*T*_MVC_ were not significant between groups H, M, and L (Table 1). The only difference between MRTD/BM in groups H and L was significant. In contrast, CMJ was significantly correlated with MRTD indices (MRTD/*T*_MVC_ and MRTD/BM):

(6) $$\begin{array}{c} \text{CMJ}=57.3+2.44\frac{\text{MRTD}}{\text{BM}}\;r=0.461;\;P=0.015,\\ \text{CMJ}=53.2+4.54\frac{\text{MRTD}}{T_{\text{MVC}}}\;r=0.444;\;P=0.020. \end{array}$$

### 3.3. Vertical Jump and Stiffness Indices

CMJ was significantly correlated with *S*_0.4_ but not with *S*_0.2_, *S*_0.6_, and *S*_0.8_:

(7) $$\begin{array}{c} \text{CMJ}=53.4+3.97\frac{S_{0.4}}{\text{BM}}\;r=0.523,\;P=0.005,\\ \text{CMJ}=57.1+4.37\frac{S_{0.4}}{T_{\text{MVC}}}\;r=0.415,\;P=0.031. \end{array}$$

**Figure 4:**
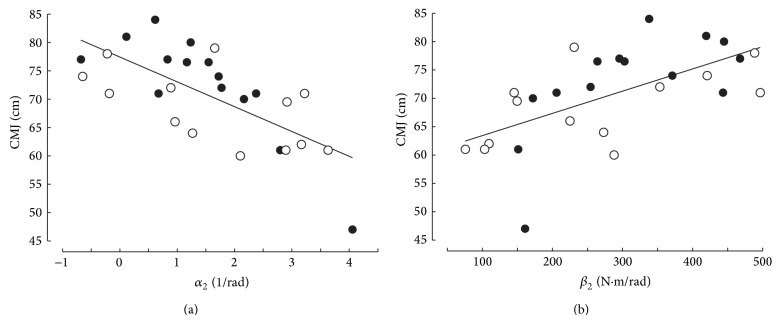
Relationship between countermovement jump (CMJ) and slope *α*_2_ and intercept *β*_2_ of the individual torque-stiffness relationships for torque ranging from 40 to 80% *T*_MVC_. Black points subject with the highest values of maximal rate of torque development (MRTD/BM). Empty circles, subjects with the lowest values of MRTD/BM.

Countermovement jump was also significantly correlated (Figure 4) with *α*_2_ (negative correlation) or *β*_2_ (positive correlation):

(8) $$\begin{array}{c} \text{CMJ}=77.4-4.4\alpha_{2}\;r=-0.690,\;P<0.001,\\ \text{CMJ}=59.5+0.039\beta_{2}\;r=0.610,\;P<0.001. \end{array}$$

### 3.4. Curvature Indices

**Figure 5:**
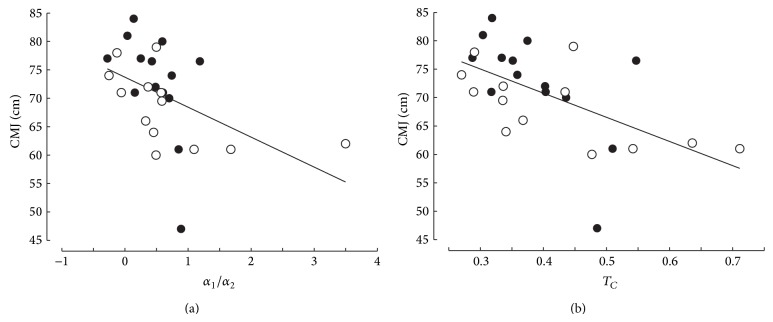
Relationship between vertical jump (CMJ) and the indices of curvature (ratio *α*_2_/*α*_1_, *T*_*C*_) of the torque-stiffness relationship. The same symbols as in Figure 4.

Ratio *α*_2_/*α*_1_ was close to 1 in group L and was independent of maximal strength indices (*T*_MVC_, *T*_MVC_/BM, *r* < 0.049) and torque development (MRTD, MRTD/BM, and MRTD/*T*_MVC_; *r* < 0.225). Interestingly, CMJ was significantly and negatively correlated with ratio *α*_2_/*α*_1_ or curvature index *T*_*C*_ (Figure 5):

(9) $$\begin{array}{c} \text{CMJ}=73.8-5.28\frac{\alpha_{2}}{\alpha_{1}}\;r=0.467;\;P=0.014,\\ \text{CMJ}=87.8-42.5T_{C}\;r=0.574;\;P=0.002. \end{array}$$

### 3.5. Multiple Regressions between CMJ, MRTD, and Stiffness Indices

The rate of torque development was not significantly correlated with the different stiffness indices (0.719 ≥ *P* ≥ 0.184) although CMJ was significantly correlated with MRTD as well as stiffness indices. As a consequence, the prediction of CMJ was improved by including torque development indices in addition to stiffness indices in multiple linear regressions. For example,

(10)  CMJ=66.4−4.02α2+1.93MRTDBM, R=0.778; n=27; P<0.001 for α2,  P=0.01 for MRTDBM,  CMJ=49.9+0.035β2+1.97MRTDBM, R=0.712; n=27; P<0.001 for  β2, P=0.017 for MRTDBM, CMJ=39.3+5.15S0.4TMVC+2.81MRTDBM, R=0.670; n=27; P=0.004 for S0.4TMVC, P=0.002 for MRTDBM, CMJ=61.6−4.66α2α1+2.15MRTDBM, R=0.617; n=27; P=0.018 for α2α1, P=0.019 for MRTDBM, CMJ=75.3−37.0TC+1.89MRTDBM, R=0.672; n=27; P=0.004 for TC, P=0.030 for MRTDBM.

These results are illustrated by separating the subjects with the highest (*n* = 14, black points) and lowest (*n* = 13, empty circles) values of MRTD/BM in Figures 4 and 5 that represent the relationships between CMJ and stiffness indices. Most of the black points are located over the regression lines in Figures 4 and 5.

## 4. Discussion

The present study was designed to relate vertical jump performance and ankle plantar flexor MTS or MRTD. The results showed that (i) CMJ was significantly correlated with the indices of torque development; (ii) CMJ was positively correlated with some indices of stiffness; (iii) the ankle plantar flexors MTS at low torque (*S*_0.4_) was significantly higher in the best jumpers that presented a torque-stiffness relationship with a plateau.

The values of stiffness in the present study were in agreement with the previous investigations on plantar flexor stiffness studied by means of fast controlled-release method [24, 25]. For example, the value of* S*100 (Table 2) that is near *T*_MVC_ in the present study was within the range of the stiffness at 100 N·m reported by Hof [24] (306 ± 39 N·m·rad^−1^) and de Zee and Voigt [25] (506 ± 72 N·m·rad^−1^). The values of CMJ in groups H and M were high when compared with CMJ data in the literature measured with the same protocol [3] or with a force platform [19, 26–28]. On the other hand, the vertical jump performance in group L was not especially low as it was similar to the average CMJ measured with the same device and protocol in the study by Vandewalle et al. [3]. The values of CMJ are higher than the values of vertical jumps without countermovement and arm swing (squat jump, SJ), especially when they correspond to the displacement of the center of mass, computed from the data of a force platform. The combination of countermovement and arm swing results in large increases in jump scores [19, 26–30].

### 4.1. MRTD versus CMJ or Stiffness Indices

The musculotendinous complex has to fulfill two contradictory requirements: to be compliant for elastic energy storage and stiff for the transmission of force. High compliance improves the possibility of elastic energy storage but lowers the rate of force development [6]. The indices of torque development were significantly correlated with vertical jump performance as observed in previous studies [7, 31–33]. Unexpectedly, the correlation coefficients between MRTD/BM or MRTD/*T*_MVC_ and stiffness indices were not significant, which could probably be explained by the prevailing importance of other factors in the rate of force development. Indeed, the rate of force development depends not only on MTS but also on muscle activation (i.e., high and fast activation) and muscle fiber types. For example, MRFD is lower during voluntary contraction than during electrical stimulation [34, 35], depends on instruction [18], and is related to muscle preactivation at the beginning of a contraction [31, 36]. The improvement in MRFD induced by training is probably the result of a better activation [37, 38]. Therefore, it is possible that fast and high muscle activations partly explained the value of MRTD/*T*_MVC_, MRTD/BM, and CMJ that was abnormally high in the best jumper when compared with the other subjects. The best values of CMJ could be partly explained not only by high and fast muscle activation but also by better timing of this activation. Indeed, according to Voigt et al. [39], the coordination of muscle activation and external loading would be necessary to optimise the output from the muscle tendon complex in stretch shortening cycles like jumping with prestretch. In addition to MTS and muscle activation, MRTD also depends on muscle fiber types. Indeed, the rate of force development by single muscle fibres in humans is similar to the difference in their maximal shortening velocities [40] that is several times higher in type IIX fibres than in type I fibres, which could partly explain why vertical jump performances are higher in subjects with high percentages of fast muscle fibers [41].

The correlation coefficients between CMJ and MRTD indices explained that the differences in MRTD indices between groups H, M, and L were not significant with the exception of the difference between MRTD/BM in groups H and L. It is possible that the importance of MRTD was attenuated by the countermovement before the jump as prestretching of the muscles during the downward phase allowed the muscles to develop a higher level of active state and force before starting to shorten [19, 29, 42].

### 4.2. CMJ and MTS

The correlation between CMJ and several stiffness indices of the plantar flexors agrees with the positive correlation between jump performance and the tendinous stiffness of the quadriceps studied by ultrasonography [7]. However, the highest coefficient of correlation between CMJ and a stiffness index of the plantar flexors in the present study (CMJ versus slope *α*_2_; *r* = 0.690; *P* < 0.001) corresponded to a coefficient of determination equal to 0.476 only. After inclusion of indices of torque development in multiple linear regressions between CMJ and stiffness indices, the coefficient of determination was largely improved (e.g., 0.605 for the multiple linear regression between CMJ, *α*_2_, and MRTD/BM). Finally, as the rate of force development depends on muscle activation and muscle fiber type, it is likely that a multiple regression between CMJ, MRTD, and stiffness indices includes the main factors determining vertical jump performance.

The stiffness indices at 40% *T*_MVC_ (*S*_0.4_) were similar in the best jumper (subject BJ in Figure 3(c)) and the worst jumper (subject WJ in Figure 3(c)) although there was a positive relationship between CMJ and *S*_0.4_/BM or *S*_0.4_/*T*_MVC_. The *T*-*S* relationship was linear in subject WJ in contrast to subject BJ (Figure 3(c)). These results suggested a possible link between CMJ and the shape of the *T*-*S* relationship in agreement with the significant negative regression between CMJ and *T*_*C*_ or ratio *α*_2_/*α*_1_. The force-length (or force-stretch) curve of isolated tendon presents a concave portion at low force (the “toe” region) followed by a “linear” segment [43]. An exponential model of the toe region corresponds to a linear stiffness-force relation in this region. A linear segment beyond the toe region corresponds to a plateau of the *T*-*S* curve. The stretch-torque curve in a fast controlled-release study of the plantar flexors and dorsi flexors in men [25, 44] was nonlinear up to torques close to MVC (i.e., the toe region extended up to MVC). Similarly, the linear relationship between torque and stiffness previously observed for the ankle plantar flexors [21, 22] when using the quick release method corresponds to an exponential toe region up to MVC. In the present study, the *T*-*S* relationship in group L could be described by a linear regression (Figure 3(b)) in agreement with the previous studies using the quick release test performed with the same device and protocol. In contrast, the force-length curve is assumed to be linear beyond 50% MVC in the ultrasonography studies of the quadriceps muscle [7], which corresponds to a plateau of the stiffness-force curve. In the present study, the relationship between stiffness and torque could not be described by a linear regression from zero to *T*_MVC_ in all the subjects (Figure 3), especially in the best jumpers. A downward inflection of the *T*-*S* relationship at high torque has been previously observed in a fast controlled-release study of the plantar flexors [24]: the torque corresponding to the demarcation point between nonlinear and linear part of the stretch-torque curve was between 0.30 and 0.70 MVC. The average demarcation point would be between 0.4 and 0.6 *T*_MVC_ in the best jumpers (Figure 3(b)) of the present study.

The results of the present study are not in favour of the hypothesis that a long compliant tendon is favorable to jump performance [10, 45], which did not mean that CMJ was not influenced by storage and recoil of elastic energy in the musculotendinous structures. Indeed, we did not compare the performance (CMJ) in a countermovement jump with the performance (SJ) in a squat jump (without countermovement) as in the study by Kubo et al. [10]. Therefore the relationships between SJ and the stiffness indices could be different from the relationships between CMJ and the stiffness indices in the present study.

## 5. Conclusions

The present study revealed that countermovement jump was significantly correlated with different indices of musculotendinous stiffness or maximal rate of torque development. Furthermore, it is suggested that differences in musculotendinous stiffness partly explain the better countermovement jump scores and that torque-stiffness relationship with a plateau at high torques is more frequent in the best jumpers.

## Figures and Tables

**Table 1:** Performances in a countermovement vertical jump (CMJ), body mass (BM), body height (BH), torque during maximal voluntary contraction (*T*_MVC_, *T*_MVC_/BM), and maximal rate of torque development (MRTD, MRTD/BM, and MRTD/*T*_MVC_) in all the subjects, in high (H), medium (M), and low (L) performers in vertical jump (CMJ). Means ± SD. *P*_HM_, *P*_HL_, and *P*_ML_ significance levels of the differences between groups L, H, and M (ANOVA; post hoc Bonferroni *t*-test).

	All (*n* = 27)	H (*n* = 9)	*P*_HM_	M (*n* = 9)	*P*_ML_	L (*n* = 9)	*P*_HL_
CMJ (cm)	70.6 ± 8.2	78.8 ± 2.5		71.8 ± 1.39		61.3 ± 6.2	
BM (kg)	78.5 ± 9.3	76.1 ± 9.0	NS	82.1 ± 10.8	NS	77.3 ± 7.8	NS
BH (cm)	182 ± 7	181 ± 8	NS	184 ± 6	NS	182 ± 7	NS
*T*_MVC_ (N·m)	111 ± 22	116 ± 29	NS	113 ± 22	NS	105 ± 15	NS
*T*_MVC_/BM (N·m·kg^−1^)	1.42 ± 0.27	1.52 ± 0.32	NS	1.38 ± 0.29	NS	1.36 ± 0.19	NS
MRTD (N·m·s^−1^)	425 ± 119	487 ± 148	NS	433 ± 100	NS	355 ± 65	NS
MRTD/*T*_MVC_ (s^−1^)	3.84 ± 0.81	4.23 ± 0.93	NS	3.86 ± 0.60	NS	3.43 ± 0.73	NS
MRTD/BM (N·m·s^−1^·kg^−1^)	5.45 ± 1.56	6.44 ± 1.99	NS	5.26 ± 0.96	NS	4.66 ± 1.09	0.041

NS: not significant.

**Table 2:** Stiffness indices (*α*_1_, *α*_2_, *β*_2_, and *S*_0.4_/BM, *S*_0.4_/*T*_MVC_), curvature indices (*α*_2_/*α*_1_, *T*_*C*_), and stiffness at 100 N·m (*S*100) in all the subjects (*n* = 27), in high (H), medium (M), and low (L) performers in vertical jump (CMJ). Means ± SD. *P*_HM_, *P*_HL_, and *P*_ML_ significance levels of the differences between groups L, H, and M (ANOVA; post hoc Bonferroni *t*-test).

	All (*n* = 27)	H (*n* = 9)	*P*_HM_	M (*n* = 9)	*P*_ML_	L (*n* = 9)	*P*_HL_
*α*_1_ (rad^−1^)	3.12 ± 1.13	2.71 ± 0.96	NS	3.46 ± 1.07	NS	3.20 ± 1.32	NS
*α*_2_ (rad^−1^)	1.56 ± 1.30	0.69 ± 0.81	NS	1.33 ± 1.25	0.041	2.64 ± 1.03	0.001
*α*_2_/*α*_1_	0.59 ± 0.73	0.30 ± 0.44	NS	0.37 ± 0.35	NS	1.11 ± 0.99	0.05
*β*_2_ (N·m·rad^−1^)	283 ± 128	361 ± 95	NS	318 ± 127	0.016	171 ± 75	0.002
*S*_04_/*T*_MVC_ (rad^−1^)	3.10 ± 0.79	3.41 ± 0.63	NS	3.36 ± 0.93	NS	2.55 ± 0.46	0.047
*S*_04_/BM (N·m·rad^−1^·kg^−1^)	4.34 ± 1.09	5.07 ± 0.92	NS	4.01 ± 0.86	NS	3.94 ± 1.16	0.05
*T*_*C*_	0.40 ± 0.11	0.40 ± 0.08	NS	0.35 ± 0.06	0.028	0.46 ± 0.15	0.029
*S*100 (N·m·rad^−1^)	417 ± 83	417 ± 81	NS	421 ± 108	NS	414 ± 65	NS

NS: not significant.

## Abbreviations

ANOVA: Analysis of variance
BH: Body height
BM: Body mass
CMJ: Countermovement vertical jump
H: High performers in vertical jump
*I*: Inertia
L: Low performers in vertical jump
M: Medium performers in vertical jump
MRTD: Maximal rate of torque development
MTS: Musculotendinous stiffness
*P*_max⁡_: Maximal anaerobic power on a cycle ergometer
*S*: Index of musculotendinous stiffness
*S*_0.2_: Index of musculotendinous stiffness at 20% of maximal voluntary torque
*S*_0.4_: Index of musculotendinous stiffness at 40% of maximal voluntary torque
*S*_0.6_: Index of musculotendinous stiffness at 60% of maximal voluntary torque
*S*_0.8_: Musculotendinous stiffness at 80% of maximal voluntary torque
SEC: Series elastic component
SJ: Squat jump
*T*: Static torque
*T*_*C*_: Empirical curvature index
*T*_MVC_: Maximal voluntary torque
*α*_1_ and *α*_2_: Slopes of the *T*-*S* relationship from 20 to 60 and 40 to 80% *T*_MVC_
*β*: *Y* intercept of the *T*-*S* relationship
Θ: Angular displacement
Θ′′: Angular acceleration.
